# Diagnosis of tuberculosis among COVID-19 suspected cases in Ghana

**DOI:** 10.1371/journal.pone.0261849

**Published:** 2021-12-28

**Authors:** Theophilus Afum, Prince Asare, Adwoa Asante-Poku, Isaac Darko-Otchere, Portia Abena Morgan, Edmund Bedeley, Diana Asema Asandem, Abdul Basit Musah, Ishaque Mintah Siam, Phillip Tetteh, Yaw Adusi-Poku, Rita Frimpong-Manso, Joseph Humphrey Kofi Bonney, William Ampofo, Dorothy Yeboah-Manu

**Affiliations:** 1 Noguchi Memorial Institute for Medical Research, University of Ghana, Accra, Ghana; 2 National Tuberculosis Control Programme, Ghana Health Service, Accra, Ghana; Indian Institute of Technology Delhi, INDIA

## Abstract

**Background:**

Tuberculosis (TB) and COVID-19 pandemics are both diseases of public health threat globally. Both diseases are caused by pathogens that infect mainly the respiratory system, and are involved in airborne transmission; they also share some clinical signs and symptoms. We, therefore, took advantage of collected sputum samples at the early stage of COVID-19 outbreak in Ghana to conduct differential diagnoses of long-standing endemic respiratory illness, particularly tuberculosis.

**Methodology:**

Sputum samples collected through the enhanced national surveys from suspected COVID-19 patients and contact tracing cases were analyzed for TB. The sputum samples were processed using Cepheid’s GeneXpert MTB/RIF assay in pools of 4 samples to determine the presence of *Mycobacterium tuberculosis* complex. Positive pools were then decoupled and analyzed individually. Details of positive TB samples were forwarded to the NTP for appropriate case management.

**Results:**

Seven-hundred and seventy-four sputum samples were analyzed for *Mycobacterium tuberculosis* in both suspected COVID-19 cases (679/774, 87.7%) and their contacts (95/774, 12.3%). A total of 111 (14.3%) were diagnosed with SARS CoV-2 infection and six (0.8%) out of the 774 individuals tested positive for pulmonary tuberculosis: five (83.3%) males and one female (16.7%). Drug susceptibility analysis identified 1 (16.7%) rifampicin-resistant tuberculosis case. Out of the six TB positive cases, 2 (33.3%) tested positive for COVID-19 indicating a coinfection. Stratifying by demography, three out of the six (50%) were from the Ayawaso West District. All positive cases received appropriate treatment at the respective sub-district according to the national guidelines.

**Conclusion:**

Our findings highlight the need for differential diagnosis among COVID-19 suspected cases and regular active TB surveillance in TB endemic settings.

## Introduction

The novel coronavirus disease 2019 (COVID-19) caused by the severe acute respiratory coronavirus 2 (SARS CoV-2) was first reported in the Wuhan suburb of Hubei province in China in early December 2019 and has since spread to all regions of the world. It was officially declared a pandemic by the World Health Organization (WHO) in March 2020 [1, 2]. Headache, fever, cough, muscle aches, tight chest or wheezing, and dyspnoea are symptoms presented by coronavirus [3]. However, these symptoms are shared by other respiratory tract infections (RTIs) including tuberculosis, pneumonia, bronchiolitis, acute bronchitis.

Tuberculosis (TB), caused by the *Mycobacterium tuberculosis* complex (MTBC) is a disease of antiquity but still remains a global health emergency [4, 5]. In 2019, an estimated 10 million people contracted TB with 1.3 million deaths accounting for the highest number of deaths caused by a single infectious disease in that year [5]. Ghana, with a TB incidence rate of 148/100,000 population per year, is ranked the 19^th^ most TB-burdened country in Africa by WHO and among the 48 most burdened TB countries in the world [6].

Undiagnosed/misdiagnosed TB is one of the major problems facing the control of TB in Ghana. Available data indicate only a third of the TB cases are reported for appropriate care. Such late/low case reports increase community transmission [5–7]. It has been portended low case detection would be exacerbated by the COVID-19 pandemic through the diversion of resources, a shift of leadership focus, stress on healthcare personnel, quarantine of healthcare personnel, and potential stigma/fear of COVID-19 [8]. There is therefore the need to find ways to maximize diagnosis of TB amidst the COVID-19 pandemic.

Ghana’s first two COVID-19 cases were diagnosed on the 12^th^ of March 2020 from individuals who had just arrived in the country by the Virology Department of the Noguchi Memorial Institute for Medical Research, (NMIMR), University of Ghana (UG) [9]. The NMIMR became the national coordinating center for COVID-19 diagnosis, using either sputum or oropharyngeal swab. Thus, we took advantage of sputum samples being transported to NMIMR by the enhanced active case finding employed by the national response team to offer a very good avenue for early case detection for TB and other respiratory infections which might be confused with COVID-19.

## Methods

### Sputum collection

Samples used for this study formed part of the enhanced national COVID-19 surveillance conducted by the Ghana health service. Sputum collected from suspected COVID-19 patients were transported to NMIMR within 5 hours of collection for COVID-19 testing. Only good quality mucopurulent sputa from patients with clinical manifestations such as cough and fever were enrolled in this study. The sputum samples were collected following national and WHO recommended guidelines to prevent the spread of COVID-19. Briefly, field staff were provided with appropriate PPE ie, lab coat, nose mask, face mask, gloves and were supplied with hand sanitizers and advised to practice frequent hand washing after each sampling while ensuring social distancing.

### MTBC detection

Sputum samples were processed using Cepheid’s GeneXpert MTB/Rif Assay [10]. This is an RT-PCR-based assay used to detect the presence of the MTBC and resistance to rifampicin in sputum samples. Samples were analyzed in pools of four and following Cepheid’s GeneXpert MTB/Rif assay manufacturer’s protocol. A negative test (MTB NOT DETECTED) meant all samples in that pool were negative for tuberculosis. A positive test (MTB DETECTED) was however decoupled and the four samples in the pool ran individually to identify the specific sample(s) positive for tuberculosis. Results were communicated to the NTP for patient follow-up on the and commencement of anti-TB drug therapy.

### Culture of MTBC positive samples and characterization

GeneXpert positive sputum samples were decontaminated by the 5% oxalate method and inoculated on two pairs of Lowenstein-Jensen media slants supplemented with glycerol and pyruvate, respectively [11]. The inoculated tubes were incubated at 37°C and observed weekly for growth for a period of 12 weeks. Isolates were confirmed as members of the MTBC by PCR targeting the insertion sequence IS*6110* [12].

### Data analysis

Chi-square test, Fischer’s exact test, or logistic regression where appropriate was used to test for statistical significance. P = values <0.05 were regarded as statistically significant at a 95% confidence level. All statistical analyses were carried out in Stata v 14.2. The GIS co-ordinates of the residential location of the cases were used to construct a graphical plot for visualization of the spread of the cases in the Greater Accra Region of Ghana using ArcMap employed in ArcGIS v10.1 [13].

### Ethical consideration

Efforts to reduce the incidence of tuberculosis to the barest minimum by 2030 may not be achieved by the upsurge of COVID-19 cases which has slowed down some of the TB control activities. To ensure early case detection of TB for efficient management and help curb the continuous spread of the pathogen, the National Tuberculosis control Program (NTP) of the Ghana Health Service together with the national coordinator of COVID-19 laboratories as a matter of emergency requested the Bacteriology department of the Noguchi Memorial Institute for Medical Research to undertake this study. Participants’ data were anonymized. All diagnosed cases were located and put on therapy per national guidelines.

## Results

### General characteristics of participants

We obtained and analyzed 774 samples from both suspected COVID-19 cases (679/774, 87.7%) and their contacts (95/774, 12.3%). Of the participants with data on gender (N = 753), 400/753 (53.1%) were males and 353/753 (46.9%) were females. All the participants came from 30 districts within 6 regions in Southern Ghana (Table 1) with the majority coming from Ayawaso (204/714, 28.6%) and Adenta Municipal (115/714, 16.1%). Sixty participants had no data on location. Nationality data was available for 765 participants of which 752 (98.3%) were Ghanaians with the remaining 13 (1.7%) being foreigners from Nigeria (n = 5), UK (n = 5), China (n = 1), France (n = 1) and Norway (n = 1).

**Table 1:** Geographical location of study participants.

Number	Region	District	Frequency N (%)
1	Greater Accra	Ayawaso	204 (28.6)
2	Greater Accra	Adenta_Municipal	115 (16.1)
3	Greater Accra	Ashaiman_Municipal	79 (11.1)
4	Greater Accra	Tema_Metropolis	75 (10.5)
5	Greater Accra	Ga_South_Municipal	41 (5.7)
6	Greater Accra	Osu_Klottey	36 (5.0)
7	Greater Accra	Ledzokuku_Krowor_Municipal (Kpeshie)	30 (4.2)
8	Greater Accra	Ablekumah	24 (3.4)
9	Greater Accra	Ga_East_Municipal	16 (2.2)
10	Greater Accra	Dangme_West_District	15 (2.1)
11	Greater Accra	Ga_Central	10 (1.4)
12	Central	Mfantseman_Municipal_District	10 (1.4)
13	Central	Awutu_Senya_East_District	9 (1.3)
14	Eastern	Lower_Manya_Krobo_District	8 (1.1)
15	Eastern	Akwatia_District	6 (0.8)
16	Eastern	Akuapim_North_Municipal_District	5 (0.7)
17	Central	Dangme_East_District	4 (0.6)
18	Central	Gomoa_West_District	4 (0.6)
19	Eastern	Suhum_Kraboa_Coaltar_district	3 (0.4)
20	Eastern	Asuogyaman_Municipal_District	3 (0.4)
21	Central	Cape_Coast_Municipal_District	3 (0.4)
22	Upper West	Sissala_East_Municipal_District	2 (0.3)
23	Western	Secondi_Takoradi_Municipal_District	2 (0.3)
24	Volta	Ketu_South_Municipal_District	2 (0.3)
25	Eastern	New_Juaben_North_District	2 (0.3)
26	Eastern	Nsawam_Adoagyiri_Municipal_District	2 (0.3)
27	Central	Komenda_Edina_Eguafo_Abirem_Municipal_D	1 (0.1)
28	Eastern	Ayensuano_District	1 (0.1)
29	Western	Ellembelle_District	1 (0.1)
30	Central	Twifu_Atii_Morkwaa_District	1 (0.1)

### SARS-CoV2 and tuberculosis infection

One hundred and eleven participants (14.3%) were confirmed by RT-PCR as having SARS-CoV-2 infection. A significant number of the COVID-19 cases were from the suspected cases (106/111, 95.5%) rather than the contact tracing group (5/111, 4.5%) p = 0.007. We observed no statistical difference (p = 0.077) between males (67/400, 16.7%) and females (43/353, 12.2%) who tested positive for SARS- CoV2. We found 6 (0.8%) out of the 774 participants to be positive for *Mycobacterium tuberculosis* infection from both the suspected COVID-19 cases (4/675, 0.6%) and contact tracing group (2/93, 2.1%) with no significant difference between the two groups (p = 0.161). Two (2/111, 1.8%) of the individuals positive for SARS-CoV-2 were found to be additionally positive for TB and were among the suspected COVID-19 group. The two contacts that were TB positive had no signs and symptoms of TB.

**Fig 1:**
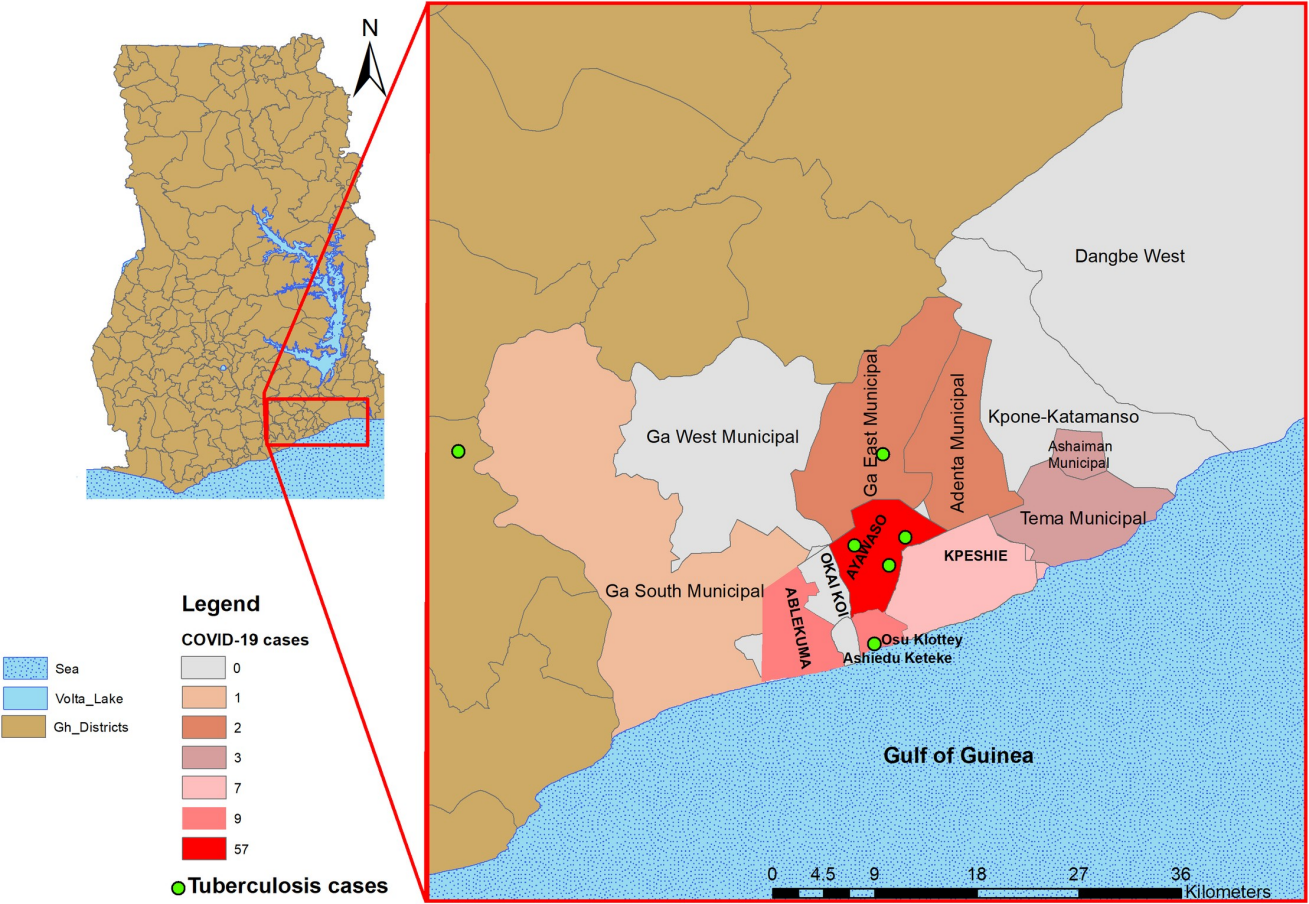
Geographical density of COVID-19 and distribution of TB cases within the sampling period.

The majority of the positive COVID-19 cases (57/104, 54.8%) were from the Ayawaso district followed by the Ablekuma and Osu Klottey districts each recording 9/104 (8.6%) positive cases (Fig 1). COVID-19 cases were found to be significantly higher among those of foreign origin compared to the Ghanaian locals (11/13, 84.6% vs. 97/752, 12.9% p<0.001). Compared to the Ghanaian locals, foreigners were 37 times more likely to be positive for COVID-19 (OR = 37, CI: 8–170). All 6 TB cases identified were, however among only Ghanaian locals mainly from the Ayawaso district (3/6, 50.0%) (Fig 1).

### Culture results

Out of the six GeneXpert positive samples, only four (4/6, 66.6%) had adequate sputum to proceed to culture stage. A culture positivity rate of 50% (2/4) was recorded. These two isolates were confirmed part of the *Mycobacterium tuberculosis* complex by IS*6110* PCR with one identified by spoligotyping to belong to lineage 4.

## Discussion

Several studies and a recent prevalence study conducted in Ghana confirms the WHO estimates that only a third of Ghana’s TB cases are reported formally for treatment [14]. Moreover, there are claims of reactivation of several respiratory diseases of the lung after infection with COVID-19. There is even the claim that active-TB disease could develop from latent infection after infection with SARS CoV-2 as evidenced in some patients with no history of TB [15]. The NTP of Ghana together with the NMIMR conducted differential TB diagnosis using sputum samples sent to the NMIMR for COVID- 19 diagnosis to ensure early case detection of TB for efficient management.

As evidenced in this study, we identified six (0.8%) TB patients among 774 suspected COVID-19 individuals with 2 being co-infected with both the MTBC and SARS CoV-2. The quality of sputum received was generally very poor (more salivary than mucopurulent) and could account for the low number of TB coinfection detected. This was expected as the samples were not primarily taken for TB diagnosis and individuals with no cough symptoms generally produce low quality sputum.

Two of the TB positive cases in this study were found as a result of the enhanced contact tracing scheme put in place by the government of Ghana to detect COVID-19 cases early to reduce community transmission. These two samples respectively showed high and medium bacterial load when analyzed with GeneXpert MTB/RIF assay indicating that there could be a high chance of community spread of TB with such high bacterial loads detected [16]. Interestingly, these two TB positive samples from contact tracing were both from the Ayawaso sub-district which our previous studies have confirmed to be a hotspot for active TB transmission [17].

This further stresses the need to conduct frequent community screening of individuals in known hotspot communities such as Ayawaso and Ablekuma sub-districts where active TB transmission has been shown to be taking place [17]. There is therefore the need for active community case search to ensure early active TB case detection to ensure efficient management to cut any potential spread.

Our findings also indicated that differential diagnoses for COVID-19 suspected patients should be encouraged as symptoms and signs of the disease are highly synonymous with other respiratory viral and bacterial infections [18]. Although the disease can have symptoms such as gastrointestinal and neurologic complications, the most common symptoms include cough, fever, and shortness of breath which overlap with symptoms common to influenza, pneumococci, and MTBC infections [19, 20]. We were limited with the data we received from participants and so was unable to carry out analysis on other comorbidities as well as other confounding variables.

The onset of COVID-19 pandemic saw a sharp decline in the number of TB tests being requested at diagnostic facilities across Ghana, including the NMIMR due to the fear of getting infected with the virus either or the stigma of being diagnosed as COVID-19 positive. This resulted in fewer cases being recorded; in the months between March and July (5 months) when the highest coronavirus cases were recorded in Ghana. Only forty-one [41] samples were received for primary diagnosis of TB compared to ninety-four [94] samples being received in the months of January and February; 2 months period. The low numbers recorded can be attributed to the reduced attention to TB as most of the health workers were primarily focused on combating COVID-19 to the neglect of other important infectious diseases [8]. A modelling study by WHO pointed to a 25% reduction in expected TB detection for 3 months as a result of COVID-19 pandemic thus derailing the Sustainable Development Goals (SDGs) of ending the TB epidemic by 2030 and WHO’s targets of 90% reduction in TB incidence and 95% death reduction by 2030 [5].

## Conclusion

The National Tuberculosis Control Programme (NTP) is adopting strategies to improve access to TB diagnosis and care with strategies such as early screening tools at OPDs, and ready access to TB treatment for home-based TB care. However, more needs to be done in the area of community education and engagement campaigns to improve early case detection and compliance to therapy to reduce negative outcomes of TB disease in Ghana. This pilot study also provided valuable information to the NTP and the health service on the need for differential diagnosis as well as strengthening surveillance of long-standing endemic infectious diseases amid the pandemic. More studies will however need to be conducted to understand better if latent TB infection progressed to active TB with COVID-19 infection or a relapse of a previous TB infection upon COVID-19 infection and the impact of co-infection on treatment outcome.
